# Ochratoxin A affects oocyte maturation and subsequent embryo developmental dynamics in the juvenile sheep model

**DOI:** 10.1007/s12550-020-00410-y

**Published:** 2020-09-29

**Authors:** Maria Elena Dell’Aquila, Shafaq Asif, Letizia Temerario, Antonella Mastrorocco, Giuseppina Marzano, Nicola Antonio Martino, Giovanni Michele Lacalandra, Bernard AJ Roelen, Augusto Carluccio, Domenico Robbe, Fiorenza Minervini

**Affiliations:** 1 Department of Biosciences, Biotechnologies and Biopharmaceutics, University of Bari Aldo Moro, Str. Prov. Casamassima Km 3, 70010 Valenzano, Bari Italy; 2 Faculty of Veterinary Medicine, University of Teramo, SP18, 64100 Teramo, Italy; 3 Department of Mathematics and Physics E. de Giorgi, University of Salento, Via per Arnesano, 73100 Lecce, Italy; 4 Department of Veterinary Sciences, University of Torino, Largo Braccini 2, 10095 Grugliasco, Torino Italy; 5 Department of Veterinary Medicine, University of Bari Aldo Moro, Str. Prov. Casamassima Km 3, 70010 Valenzano, Bari Italy; 6 Department of Clinical Sciences, Faculty of Veterinary Medicine, Utrecht University, Uppsalalaan 8, 3584 CT Utrecht, The Netherlands; 7 Institute of Sciences of Food Production (ISPA), National Research Council of Italy (CNR), Via G. Amendola 122/O, 70125 Bari, (BA) Italy

**Keywords:** Ochratoxin A, Juvenile sheep oocyte, In vitro maturation, In vitro fertilization, Bioenergetic/oxidative status, Time-lapse embryo monitoring

## Abstract

The genotoxic and nephrotoxic mycotoxin Ochratoxin A (OTA) has also been reported to have adverse effects on oocyte maturation and embryo development. Previous studies on the effects of OTA on female fertility have used micromolar concentrations, but no information is available to date on effects in a more relevant nanomolar range. This study used a juvenile sheep model to evaluate the effects of oocyte exposure to low levels of OTA on maturation, fertilization, and embryo development. Further, it was investigated whether different mechanisms of action of OTA could be responsible for varying toxic effects at different levels of exposure. Cumulus-oocyte-complexes (COCs) were exposed to 10 μmol/L–0.1 nmol/L OTA during in vitro maturation and evaluated for cumulus viability, oocyte maturation, and bioenergetic/oxidative status. COCs were subjected to in vitro fertilization, embryo culture, and embryo quality assessment via morphology, viability, bioenergetic/oxidative status, and time-lapse monitoring. At micromolar concentrations, OTA induced cytotoxic effects, by reducing cumulus expansion and oocyte maturation. OTA altered temporospatial dynamics of zygote pronuclear formation and embryo morphokinetics. Blastocysts, even morphologically normal, were found to undergo collapse events, which were probably related to boosted blastocyst mitochondrial activity. At nanomolar concentrations, OTA did not affect COC morpho-functional parameters, but impaired oocyte ability to prevent polyspermy and increased blastocyst apoptosis. In conclusion, in the female germ cell, cytotoxic nonspecific effects characterize OTA-induced toxicity at high exposure levels, whereas fine tuning-mode effects, not associated with altered cell viability and integrity, characterize OTA toxic action at low levels.

**Electronic supplementary material:**

The online version of this article (10.1007/s12550-020-00410-y) contains supplementary material, which is available to authorized users.

## Introduction

Ochratoxin A (OTA) is a naturally occurring mycotoxin produced, as a secondary metabolite, by several fungi of *Aspergillus* and *Penicillium* genera (Malir et al. 2016). Because of its ubiquitous presence in a variety of human foodstuffs (EFSA 2020) and animal feed (Duarte et al. 2011), it exerts various hazardous effects on human and animal health (Malir et al. 2016; Kőszegi and Poór 2016). OTA has a wide range of toxicological effects, including nephrotoxicity, teratogenicity, mutagenicity, genotoxicity, carcinogenicity, immunotoxicity, neurotoxicity, and hepatotoxicity, and it has been classified into group 2B as possibly carcinogenic to human (EFSA 2020).

The mechanisms of action of OTA are highly complex. Inhibition of protein synthesis and energy production, induction of oxidative and nitrosative stress, DNA adduct formation, as well as apoptosis/necrosis induction, and cell cycle arrest have been reported (Kőszegi and Poór 2016; Tao et al. 2018). The association between oxidative stress and the loss of mitochondrial membrane potential with apoptosis produced by OTA was noted in a range of cell types in vitro (EFSA 2020). Proteomic approaches have also been used to assess factors involved in OTA toxicity. Notable are the enhanced expression of proteins involved in the perturbation of mitochondrial electron transport, inhibition of protein synthesis, induction of stress response and cell death (Shen et al. 2013; EFSA 2020). OTA was shown to interfere with connexin-mediated intercellular communication in the absence of cytotoxicity (EFSA 2020). OTA might also promote tumour formation via interference with microtubule dynamics and mitotic spindle formation, resulting in apoptosis or interruption of mitosis with cytogenetic abnormalities (EFSA 2020). Up to now, there is insufficient evidence to support either direct or indirect DNA damage in OTA carcinogenesis (EFSA 2020). The genotoxic power of OTA could be caused either by direct covalent binding to DNA or as a consequence of OTA-induced oxidative damage (Gupta et al. 2017).

In humans, the relatively long half-life of OTA (Studer-Rohr et al. 2000), its ability both to cross the placental passage, and its transfer into breast milk (Biasucci et al. 2011; Muñoz et al. 2010; Gareis et al. 1988) are well known. However, no studies are available on its effects on female fertility, and most studies have used animal models. Although OTA has been reported to induce reproductive and developmental toxicity in animals (Malir et al. 2014; Gupta et al. 2017), few studies have been published to date on direct effects of OTA exposure on female gametes. Huang and Chan (2014) were the first to investigate the effects of OTA on mouse oocyte in vitro maturation (IVM), in vitro fertilization (IVF), and subsequent development. OTA significantly impaired oocyte maturation, and decreased IVF rates and embryonic development in vitro. A subsequent comparative study between mouse and porcine oocytes confirmed a significant reduction of the maturation rate in vitro in both species, but porcine oocytes were more sensitive to OTA than mouse oocytes (Lu et al. 2018). A recent study (Lan et al. 2019), using high-throughput technologies, showed that OTA exposure altered the expression of multiple genes in oocytes, indicating its molecular effects on oocyte maturation. OTA adversely affected porcine oocyte polar body (PB) extrusion by delaying CDC2-mediated cell cycle progression, and also disrupted meiotic spindle formation by altering phosphorylated MAPK expression. RNA-seq screening demonstrated that an OTA-induced aberration of oocyte mitochondria distribution and oxidative phosphorylation defects, which then caused oxidative stress, followed by early apoptosis and autophagy (Lan et al. 2019). Another multitechnological study using a mouse model (Jia et al. 2019) recently showed that OTA decreased oocyte maturation and fertility by inducing oxidative stress and epigenetic changes. In detail, OTA intraperitoneal injection induced ovarian dysfunction with decreased offspring number. OTA exposure disrupted spindle formation and chromosome alignment, preventing first PB extrusion. In addition, OTA caused oocyte apoptosis as a result of enhanced oxidative stress during meiosis. Oocytes exposed to OTA also exhibited damaged mitochondria and insufficient energy supply, leading to meiotic failure. Epigenetic modifications were also affected in mouse oocytes (Jia et al. 2019). Oocyte preincubation with OTA during IVM was shown to have negative long-term effects on embryo quality and viability. Huang and Chan (2014) reported that preincubation of oocytes with OTA during IVM increased post-implantation embryonic resorption and reduced the blastocyst total cell number. Particularly, the number of cells of the inner cell mass was reduced more than that of the trophoblast. The same authors reported that apoptosis, via p53-p21 and caspase-3-dependent regulatory mechanisms, increased in blastocysts derived from the OTA-preincubated oocytes compared with the untreated group (Huang and Chan 2014).

Unfortunately, all studies published to date on oocyte maturation and developmental competence had used very high concentrations of OTA in a micromolar range, which raises questions concerning the practical relevance of these findings. While micromolar concentrations can be attained only under experimental conditions, the nanomolar range is of relevance under real life conditions (Gekle et al. 2005), as concluded from OTA levels detected blood serum of humans (Studer-Rohr et al. 2000) and animals (Blank et al. 2003). An assessment of OTA in a concentration range going down to the nanomolar level would therefore be important to obtain more complete information. Moreover, in other cell systems, OTA has been reported to induce nonspecific cytotoxic effects at high doses (> 1 μmol/L) but specific cell signalling-mediated effects at low doses (< 1 μmol/L; Gekle et al. 2005). Therefore, the present study aimed at determining the effects of oocyte exposure to OTA at a nanomolar to micromolar concentration range on nuclear and cytoplasmic maturation, on fertilization, and on embryo development and quality. These results should be the basis for an assessment of possible differences between mechanisms responsible for toxic effects at different concentration levels.

## Materials and methods

### Chemicals

OTA (O1877-5 mg; ≥ 98%; molecular mass: 403.81 g/mol) and all chemicals for in vitro cultures and analyses were purchased from Sigma-Aldrich (Milan, Italy) unless otherwise indicated. OTA stock solution was obtained by dissolving 5 mg OTA with 5 ml of HPLC grade methanol (MeOH; ≥ 99.9%), as vehicle, to obtain a concentration of 1 mg/mL. Then, 2 mL of this solution were diluted in 3 mL of MeOH to obtain a final concentration of 1 mmol/L. An OTA solution at the concentration of approximately 10 μg/μL was prepared and spectrophotometrically tested (*ε* = 6330 cm^2^/mmol at *λ* = 332 nm in methanol) to determine the exact concentration. Aliquots were stored at − 20 °C until use.

### Collection of ovaries and oocytes

Slaughterhouse ovaries were (Fin. Sud Import s.r.l.; Conversano, Bari) obtained from juvenile ewes (less than 6 months of age) subjected to routine veterinary inspection in accordance with the specific health requirements stated in Council Directive 89/556/ECC. After transport, within 2–4 h after slaughter, ovaries were processed at the laboratory by using the slicing procedure (Martino et al. 2012) to obtain COCs collected in phosphate buffered saline (PBS) solution. For further in vitro culture, only those COCs with intact cumulus cell layers and homogeneous cytoplasm were used.

### In vitro maturation

In vitro maturation (IVM) was performed as reported previously (Mastrorocco et al. 2019). IVM medium composition is reported in Online Resource 1. COCs were placed in four-well dishes (Nunc Intermed, Roskilde, Denmark) containing 400 μL of IVM culture medium perwell of a 4-well dish, covered with paraffin oil and cultured in vitro for 24 h at 38.5 °C under 5% CO_2_ in air. On the day of experiments, serial log-dilutions of OTA stock solution with IVM medium were made to cover a concentration range from 10 to 0.0001 μmol/L, according to the requirements of the experimental design. The highest concentration was selected on the basis of published data for mice (Huang and Chan 2014) while the nanomolar concentration was chosen as corresponding to OTA blood levels in sheep, after oral ingestion of OTA-contaminated feed (Blank et al. 2003). IVM medium containing 1% MeOH was used as vehicle control. For each set of experiments, at least two runs/replicates were performed, where a run/replicate was a group of 20–25 COCs cultured for IVM in one well of a 4-well Nunc plate.

### Terminal deoxynucleotidyl transferase-mediated dUTP Nick-End Labeling (TUNEL) assay

Cumulus cells collected in MPM with 20% fetal calf serum (FCS) were spun down at 300*g* for 5 min and analyzed with the Click-iT® Plus TUNEL Assay, Molecular probe Life Technology, code: C10617, according the manufacturer’s instructions. Briefly, cells were fixed in 4% paraformaldehyde in PBS for 15 min at room temperature, washed with PBS, and subsequently permeabilized for 20 min with 0.5% Triton X-100. Then, cells were washed twice with deionized water, placed in 50 μl drops of TUNEL reagent, and incubated in the dark for 1 h at 37 °C in a humidified chamber. After that, cells were washed with 3% bovine serum albumin (BSA) in PBS and stained with 2.5 μg/ml Hoechst 33258 in 3:1 (v/v) glycerol/PBS, mounted on slides and kept at 4 °C in the dark until observations performed using an E-600 Nikon fluorescent microscope equipped with a 365 nm excitation filter.

### In vitro fertilization (IVF) and in vitro embryo culture

In vitro fertilization was performed in Synthetic Oviductal Fluid (SOF) medium (Mastrorocco et al. 2019; Tervit et al. 1972) supplemented with 2% oestrous sheep serum and 1 μg/mL heparin (Martino et al. 2016). SOF medium composition is reported in Online Resource 1, and oestrous sheep serum was prepared following the procedure by Barrera et al. (2018). Oocytes were cultured for 22 h with frozen-thawed semen (1.5 × 10^6^ spermatozoa/mL) at 38.5 °C and under a 5% CO_2_, 90% N_2_ atmosphere in 4-well dishes. Presumptive zygotes were denuded by gently pipetting using finely drawn glass pipettes and cultured for 7 days in 4-well dishes in SOF with essential and non-essential aminoacids (SOF-aa; Mastrorocco et al. 2019; Walker et al. 1996; Online Resource 1) and 0.4% BSA under mineral oil, in maximum humidified atmosphere with 5% CO_2_, 90% N_2_ at 38.5 °C. Embryos were examined at day 7 and classified according to expansion and hatching status (Martino et al. 2016).

### Sperm-free IVF

Sperm-free IVF experiments were performed to assess whether polyspermy or parthenogenesis may occur after OTA exposure. Analogous procedures as described above were used, except that sample semen was omitted in IVF plates.

### Time lapse monitoring (TLM) and morphokinetic parameters

TLM of embryos cultured in imaging dishes was performed using a commercial TLM system (PrimoVision Time Lapse Imaging System; Vitrolife) which employed Hoffman (~ 1 px mm ^−1^) modulation optics. The embryos were only illuminated during image acquisition with green light-emitting diode (LED, 550 nm, 100 ms per image) to reduce stress. Image acquisition (black and white, 2560 × 1920 pixels) started 6 h after IVF, every 10 min during 8 days of in vitro culture. Every 60 min, the images were generated on eleven focal planes and Primovision software was used to analyse the images. Development was analyzed using criteria for human embryos (Meseguer et al. 2011; Hojnik et al. 2016), modified and adapted to the time course of ovine embryos (Martino et al. 2016). Cleavage timings to 2-, 3-, 4-, 5,- and 8-cell stages (*t*2, *t*3, *t*4, *t*5, and *t*8 respectively) were recorded. Variables in cell cycle duration were defined as cc2 (*t*3–*t*2), the time spent as a 2-cell embryo and s2 (*t*4–*t*3), indicating the degree of synchrony of the two divisions from the 2- to 4-cell stage (i.e., time spent as a 3-cell embryo). Patterns of embryo development were identified according to Cruz et al. (2012) and Hojnik et al. (2016). Time of blastocyst identification (*t*B), blastocyst expansion (*t*Bexp), and cell protrusion through the zona pellucida (*t*Bcp) were also recorded.

### Staining for mitochondria and reactive oxygen species (ROS)

Oocytes and blastocysts were washed with PBS with 3% BSA/PBS and incubated with MitoTracker Orange CMTM Ros (280 nmol/L; Molecular Probes) for 30 min at 38.5 °C under 5% CO_2_ (Martino et al. 2012; Somoskoi et al. 2015). Structures were stained with the MitoTracker Orange. Negative controls were further incubated (5 min) in the presence of 5 μmol/L of the mitochondrial membrane potential (Delta Psi)-collapsing uncoupler carbonyl cianide 3-chloro phenylhydrazone (CCCP; Molecular Probes; Valentini et al. 2010). Structures were subsequently washed in PBS with 0.3% BSA and incubated for 15 min with 3% BSA/PBS containing 10 μmol/L 2′,7′- dichlorodihydrofluorescein diacetate (H2DCF-DA), at 38.5 °C under 5% CO_2_, to detect the dichlorofluorescein (DCF) and localize intracellular sources of ROS (Yang et al. 1998). After a washing step with PBS, samples were fixed overnight at 4 °C in 2% paraformaldehyde in PBS (Ambruosi et al. 2011). Exposure to light was avoided during all procedures.

### Nuclear chromatin evaluation

Oocytes and embryos were fixed 2% paraformaldehyde solution in PBS and stained with Hoechst 33258 (2.5 μg/mL) in 3:1 (v/v) glycerol/PBS and mounted on microscope slides with coverslips, sealed with nail polish and kept at 4 °C in the dark until observation using an epifluorescence microscope (Nikon Eclipse 600; × 400 magnification) equipped with a B-2A (346 nm excitation/460 nm emission) filter. Oocytes were classified as germinal vesicle (GV), metaphase to telophase I (MI to TI), MII with the 1st PB extruded, or as activated or degenerated (Martino et al. 2012). Fertilization assessment was performed as described (Tessaro et al. 2015). Normally fertilized oocytes contained two pronuclei (2PN) and two polar bodies (2PB). The occurrence of only one PN (1PN), a sperm head (SH) that failed chromatin decondensation, and 2PB were considered as uncompleted pronuclear formation. The occurrence of a second meiotic spindle (MII) with a PB indicated unfertilized matured oocytes. Cells with more than two pronuclei (> 2PN) resulted from either polyspermic fertilization or parthenogenetic activation. For zygote classification according to pronuclear morphology, the criteria for six categories (PN0– PN5) were used as described by Heras et al. (2017): PN0, decondensing sperm head and meiosis II finished; PN1, presence of two small pronuclei; PN2, larger, more centered pronuclei; PN3, maximum size pronuclei; PN4, pronuclei with fibrillary aspect. Embryos were classified according to their number of nuclei and morphology. The formation of micronuclei and lobulated nuclei was considered as signs of chromatin damage.

### Assessment of mitochondrial distribution pattern and intracellular ROS localization

Oocytes at blastocysts were examined at × 600 magnification in oil immersion with a Nikon C1/TE2000-U confocal laser scanning microscope (CLSM); 25 optical series were made per oocyte and embryo with a step size of 0.45 μm. MitoTracker Orange CMTM Ros was imaged using a helium/neon laser at 543 nm and the G-2A filter (551 nm excitation and 576 nm emission), while DCF was imaged with an argon ion laser at 488 nm and the B-2A filter (495 nm excitation and 519 nm emission) The mitochondrial distribution pattern was evaluated on the basis of previous studies. Homogeneously distributed mitochondria indicated low energy cytoplasmic condition; perinuclear and subplasmalemmal mitochondria were considered indicative of healthy cytoplasmic condition (P/S); irregularly distributed of mitochondria were classified as abnormal (Martino et al. 2012; Martino et al. 2013; Somoskoi et al. 2015). Oocytes and embryos where mitochondria and ROS were overlapping were considered as normal (Martino et al. 2012, 2013; Somoskoi et al. 2015).

### Quantification of MitoTracker Orange CMTM Ros and H2DCF-DA fluorescence intensity

For the oocytes, MitoTracker and DCF fluorescence intensities were measured at the equatorial plane using the EZ-C1 Gold Version 3.70 image analysis software on the whole cytoplasmic areas. For the blastocysts, all the 25 acquired focal planes were used. Sample signals were expressed as arbitrary densitometric units (ADU). Parameters related to fluorescence intensity were maintained at constant values for all measurements (Martino et al. 2012, 2013, 2016; Dell’Aquila et al. 2014; Somoskoi et al. 2015).

### Mitochondria-ROS colocalization analysis

Colocalization analysis of mitochondria and ROS was performed with the EZ-C1 Gold Version 3.70 software. Degree of mitochondria/ROS colocalization was reported as the overlap coefficient. Mitochondria/ROS co-localization was considered as indicating healthy, normal oocytes and embryos (Martino et al. 2013, 2016; Dell’Aquila et al. 2014; Somoskoi et al. 2015).

### Statistical analysis

The proportions of oocytes showing the different chromatin configurations and mitochondria distribution patterns, the proportions of cleaved embryos and blastocysts, and the percentages of cumulus cells showing DNA fragmentation were compared among groups by Chi Square test. The Yates’ correction was adopted according to the sample size. Morphokinetic parameters were compared between groups by unpaired *t* test. The fluorescence values of mitocondrial function, ROS, and colocalization were plotted by box and whisker plot that showed the median, 1st and 3rd quantile, the minimum, and maximum by using SigmaPlot software. The fluorescence values were compared by one-way ANOVA Kruskal-Wallis non-parametric test. Differences with *p* < 0.05 were considered to be statistically significant.

## Results

### Effects on cumulus viability and oocyte maturation

At 10 μmol/L, OTA reduced cumulus expansion and oocyte nuclear maturation rates, and increased percentages of apoptotic cumulus cells (*p* < 0.0001; Table 1). At 1 μmol/L, it significantly reduced the cumulus expansion rate (*p* < 0.0001) even if it did not affect the cumulus cell apoptotic index. At this concentration, OTA reduced the maturation rate and increased the percentage of oocytes found at the GV stage (*p* < 0.01). At 0.1 nmol/L, no effects on cumulus expansion and apoptosis or nuclear maturation were observed. Representative photomicrographs of OTA-induced inhibition of cumulus expansion and apoptosis are provided in Online Resource 2 whereas Online Resource 3 shows OTA-dependent oocyte chromatin configurations. At all tested OTA concentrations, chromatin configuration of specific meiotic stages had normal appearance. Abnormal configurations consisted in chromatin dispersion into multiple groups.

**Table 1:** Effects of in vitro exposure to OTA during IVM on oocyte meiotic progression and maturation

OTA concentration (μmol/L)	No. of cultured COCs	Cumulus expansion rate *N* (%)	No. (%) of apoptotic/examined cells	No. of evaluated oocytes	Nuclear chromatin configurations *N* (%)			
					GV	MI to TI	MII	Abnormal
0	342	91/171 (53) a	5/160 (3) a	307	97 (32) a	32 (10)	131 (43) a	47 (15)
10	333	28/169 (17) e	56/160 (35) e	281	106 (38)	41 (15)	75 (27) e	59 (21)
1	269	33/109 (30) e	9/160 (5)	232	103 (44) c	32 (14)	60 (26) e	37 (16)
0.0001	151	90/151 (53)	5/160 (3)	127	39 (31)	19 (15)	44 (35)	25 (20)

For each experimental condition, 6 to 16 replicates were performed. A replicate included 20–25 COCs cultured for IVM in one well of a 4-well plate. Chi Square test: Comparisons OTA-exposed versus vehicle control (1%MeOH): a, c = *p* < 0.01; a, e = *p* < 0.0001

### Effects on oocyte bioenergetic and oxidative status

**Fig. 1:**
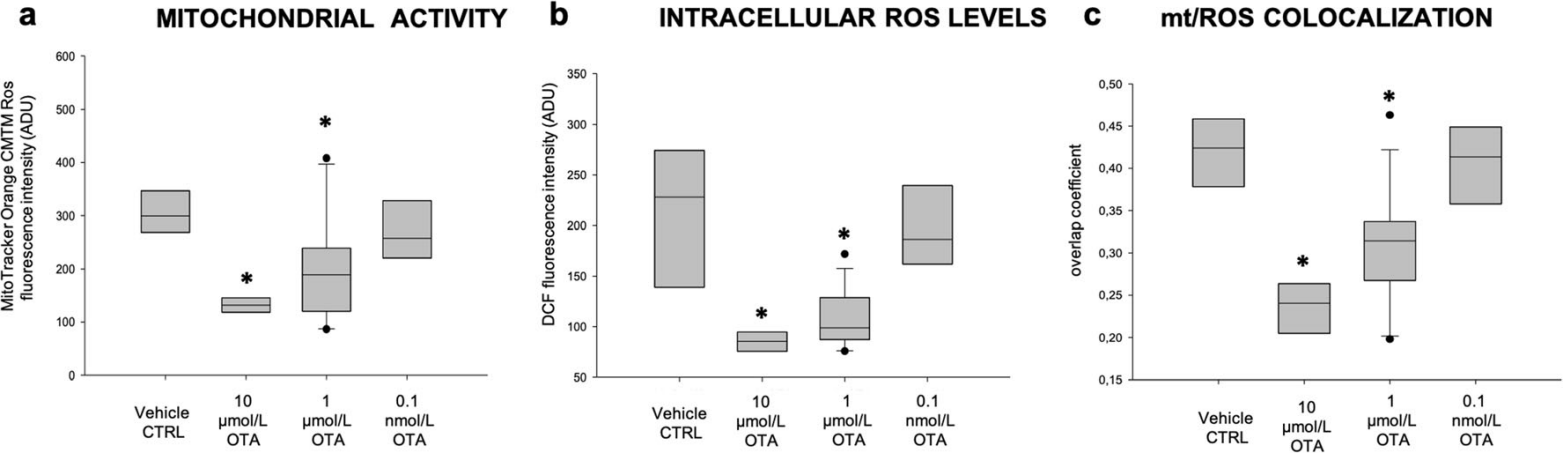
Boxplot presentation of data on mitochondrial activity, intracellular reactive oxygen species (ROS) levels, and mitochondria/ROS colocalization in MII oocytes cultured in the absence of OTA or in presence of 10 μmol/L, 1 μmol/L, and 0.1 nmol/L OTA. Data are referred to MII oocytes in Table 1. Mitochondrial activity and ROS levels are expressed as MitoTracker Orange CMTM Ros (panel **a**) and DCF (panel **b**) fluorescence intensity in arbitrary densitometric units (ADU); mitochondria/ROS colocalization is expressed as overlap coefficient (panel **c**). Oocytes matured in thepresence of 10 μmol/L and 1 μmol/L OTA showed significantly reduced mitochondrial activity, intracellular ROS levels, and mitochondria/ROS colocalization. No effects were noticed on oocytes matured in the presence of 0.1 nmol/L OTA. One-way ANOVA Kruskal-Wallis non-parametric test, comparisons OTA-exposed vs control: *p < 0.05

**Fig. 2:**
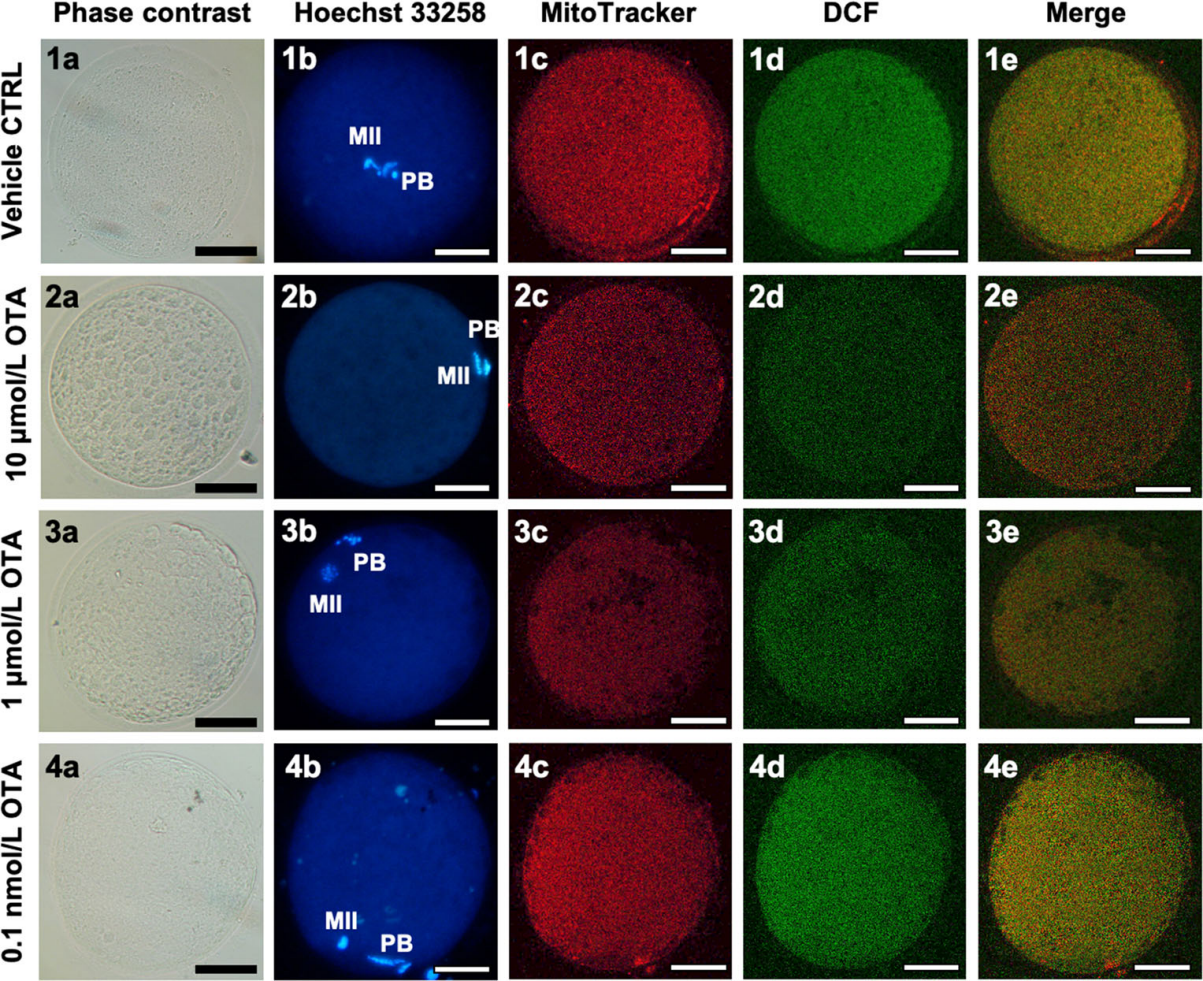
Photomicrographs showing representative images of a control oocyte (1) and of oocytes exposed to 10 μmol/L (2), 1 μmol/L (3), and 0.1 nmol/L OTA (4), respectively. Images are referred to MII oocytes in Table 1. Corresponding phase-contrast images showing cell morphology (column a), epifluorescence images showing nuclear chromatin configuration (column b: Hoechst 33258), and confocal images showing mitochondrial distribution pattern and activity (column c: MitoTracker Orange), intracellular ROS localization and levels (column d: DCF) and mitochondria/ROS colocalization (column e: Merge). Confocal images were taken at the oocyte equatorial plane. Decreased mitochondrial activity and intracellular ROS levels, expressed as decreased MitoTracker (2c and 3c vs. 1c) and DCF fluorescent intensity (2d and 3d vs. 1d) are visible in oocytes exposed to 10 μmol/L and 1 μmol/L OTA. Scale bars represent 40 μm. OTA, Ochratoxin A; DCF, dichlorodihydrofluorescein; ROS, reactive oxygen species

**Fig. 3:**
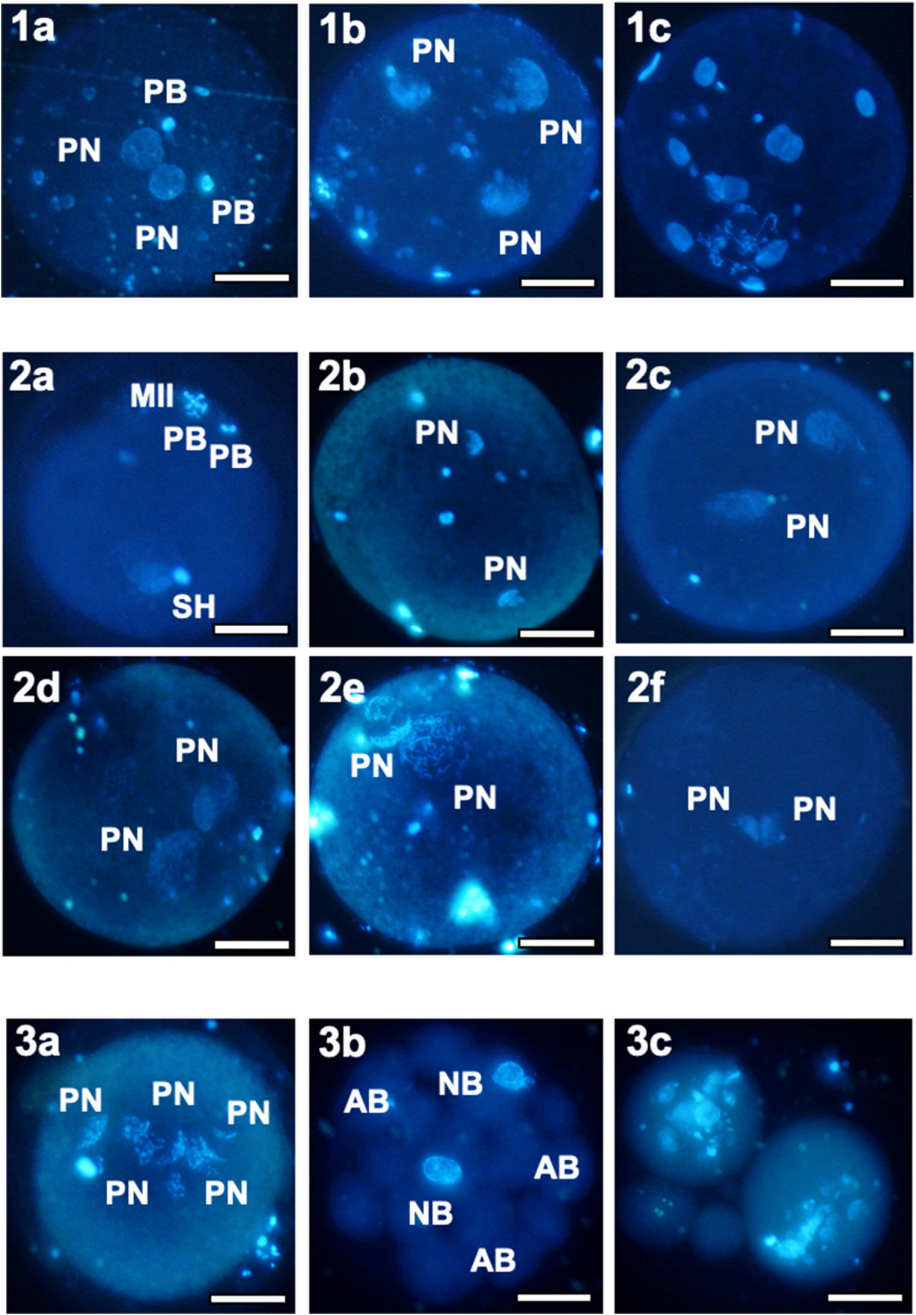
Photomicrographs showing typical nuclear chromatin configuration of normally and abnormally fertilized oocytes (lane 1), pronuclear development and migration (lane 2), and parthenogenetically activated oocytes (lane 3): (1a) normal zygote with 2 PN and 2 PB, obtained after IVM in control condition and IVF; abnormally fertilized oocytes with 3 (1b) or more than 2 PN (1c), obtained from oocytes cultured in presence of OTA and IVF. (2a) Stage 0: decondensing sperm head and meiosis II completed with the second polar body extruded and metaphase chromosomes starting to decondense; (2b) Stage 1: two small pronuclei are forming from the decondensed DNA of maternal and paternal origin; (2c) Stage 2: the pronuclei are increasing in size and start to migrate toward the center; (2d) Stage 3: the pronuclei reach their maximum size and are in apposition; (2e) Stage 4: pronuclei are in apposition and their chromatin displays fibrillary aspect; (2f) PNs with abnormal morphology, showing apposition but minimum size. (3a) Multinucleated zygote showing more than 2 PN; (3b) cleaved embryos with two nucleated (NB) and many anucleated blastomeres (AB); (3c) cleaved embryos with dispersed chromatin in each blastomere. Images are referred to zygotes in Table 3. Scale bars represent 40 μm. MII, Metaphase II; PB, polar body; PN, pronuclei; NB, nucleated blastomere; AB, anucleated blastomere

The percentage of oocytes showing heterogeneous perinuclear and subcortical (P/S) pattern, indicating cytoplasm maturity and competence, was significantly reduced after exposure to 10 μmol/L OTA (*p* < 0.0001; Table 2). This parameter was not altered at the other tested concentrations. Part of the oocytes underwent quantification and colocalization analysis. Mitochondrial membrane potential and intracellular ROS levels were significantly reduced after culture in the presence of 10 μmol/L and 1 μmol/L OTA compared with controls (Fig. 1, panels a, b; *p* < 0.05). As well, mitochondria/ROS colocalization was significantly reduced at these OTA concentrations (Fig. 1, panel c; *p* < 0.05). Instead, at 0.1 nmol/L, OTA did not affect any biomarker of oocyte bioenergetic/oxidative status. In Fig. 2, representative photomicrographs of a control oocyte (lane 1) and oocytes exposed to examined OTA concentrations (lanes 2–4) are shown. Oocytes exposed to OTA at 10 μmol/L and 1 μmol/L had lower MitoTracker and DCF fluorescence intensities (Fig. 2, (2c, d) and (3c, d)), as compared both with controls and with oocyte exposed to 0.1 nmol/L OTA.

**Table 2:** Effects of in vitro exposure to OTA during IVM on oocyte mitochondrial distribution pattern

OTA concentration (μmol/L)	No. of evaluated MII oocytes (*)	Mitochondria distribution pattern *N* (%)	
		Perinuclear/subcortical	Small aggregates
0	85	48 (56) a	37 (44) a
10	68	15 (22) e	53 (78) e
1	36	14 (39)	22 (61)
0.0001	35	14 (40)	21 (60)

(*) Data are referred to MII oocytes from Table 1

Chi Square test: Comparisons OTA-exposed versus vehicle control (1%MeOH): a, e = *p* < 0.0001

### Effects on oocyte fertilization

At any tested concentration, OTA exposure during maturation significantly affected oocyte fertilizability (Tables 3 and 4). In detail, it significantly reduced the normal fertilization rate at 10 μmol/L (*p* < 0.001), 1 μmol/L, 1 nmol/L, and 0.1 nmol/L (*p* < 0.05, Table 3) expressed as the percentage of oocytes showing two pronuclei (PN). In oocytes exposed to 0.1 nmol/, OTA significantly increased the percentage of abnormally fertilized oocytes showing > 3 PNs (Table 3; *p* < 0.05). This trend was also observed at 10 μmol/L, even if it was not quite statistically significant (*p* = 0.0915). In order to investigate OTA effects on the fertilization mechanisms, the obtained zygotes were analyzed for their PN size and position. OTA significantly reduced the percentage of zygotes at the PN3 stage at 10 μmol/L (*p* < 0.01), 0.1 μmol/L (*p* < 0.01), and 0.01 μmol/L (*p* < 0.05, Table 4). This indicates that it affected the PN ability to reach its maximum size and apposition. Finally, at 0.1 μmol/L, it also significantly increased the rate of 2PN zygotes with a specific abnormal morphology, i. e., found PN apposition but minimum size (*p* < 0.01, Table 4). In Fig. 3, representative micrographs of 2PN (1a) and more than 2PN (1b, 1c) obtained after IVM culture in the presence of OTA and IVF and representative images of zygotes with the different PN morphologies (2a-2f) corresponding to the different stages of pronuclear development obtained after IVM culture in the presence of OTA and IVF, are displayed. No differences we found in parthenogenetic activation rate between OTA-exposed and control oocytes (Table 5). Mostly, parthenogenetic activation occurred as multinucleated zygotes, showing with 2 to 10 PNs, or as irregularly cleaved embryos with nucleated, anucleated, or multinucleated blastomeres (Fig. 3, (3a–3c)). These data indicate that the presence of OTA during maturation did not lead to parthenogenetic activation.

**Table 3:** Effects of in vitro exposure to OTA during IVM on oocyte fertilization

OTA concentration (μmol/L)	No. of cultured COCs	No. of evaluated oocytes	Nuclear chromatin configurations *N* (%)	
			Normally fertilized oocytes with 2PN	Abnormally fertilized oocytes (> 2PNs)
0	265	173	53 (30.6) a	23 (13.3) a
10	140	116	12 (10.3) d	25 (21.5) (*)
1	178	136	24 (17.6) b	24 (17.6)
0.1	131	101	22 (21.7)	15 (14.8)
0.01	139	106	23 (21.7)	12 (11.3)
0.001	153	121	23 (19.0) b	17 (14.0)
0.0001	232	168	34 (20.2) b	39 (23.2) b

For each experimental condition, 5 to 9 replicates were performed.

Chi Square test: Comparisons OTA-exposed versus vehicle control (1%MeOH): a, b = *p* < 0.05; a, d = *p* < 0.001. (*) *p* = 0.0915

**Table 4:** Effects of in vitro oocyte exposure to OTA during IVM on pronuclear development and migration

OTA concentration (μmol/L)	N° of evaluated zygotes (*)	Stages of pronuclear development and migration N (%)					Abnormal chromatin configurations
		Stage 0 (decondensing sperm head)	Stage 1 (2PNs with small size)	Stage 2 (growing and migrating PNs)	Stage 3 (maximum PN size and apposition)	Stage 4 (2PNs with fibrillar chromatin)	
0	53	3 (5.7)	3 (5.7)	8 (15.1)	32 (60.4) a	2 (3.8)	5 (9.4) a
10	12	2 (16.7)	2 (16.7)	2 (16.7)	1 (8.73) c	3 (25)	2 (16.7)
1	24	0 (0)	1 (4.2)	3 (12.5)	11 (45.8)	4 (16.7)	5 (20.8)
0.1	22	0 (0)	1 (4.5)	7 (31.8)	5 (22.7) c	1 (4.5)	8 (36.4) c
0.01	23	1 (4.3)	0 (0)	6 (26.1)	7 (30.4) b	5 (21.7)	4 (17.4)
0.001	23	0 (0)	1 (4.3)	4 (17.4)	11 (47.8)	3 (13)	4 (17.4)
0.0001	34	2 (5.9)	1 (2.9)	4 (11.8)	14 (41.2)	6 (17.6)	7 (20.6)

(*) Data are referred to zygotes obtained in Table 3. Chi Square test: Comparisons OTA-exposed versus vehicle control (1%MeOH): a, b *p* < 0.05; a, c = *p* < 0.01

**Table 5:** Effects of in vitro exposure to OTA during IVM on oocyte parthenogenetic activation under sperm-free IVF

OTA concentration (μmol/L)	No. of cultured COCs	Cumulus expansion rate *N* (%)	No. of evaluated oocytes	Patterns of parthenogenetic activation *N* (%)		Total parthenogenesis *N* (%)	Metaphase II *N* (%)	Total matured (metaphase II+ parthenogenetic) *N* (%)
				Multinucleated zygotes	Cleaved embryos			
0	81	54 (67)	78	8 (10)	6 (7.7)	14 (18)	32 (41)	46 (59)
10	40	5 (12.5)	39	4 (10)	1 (3)	5 (13)	19 (49)	24 (60)
0.0001	80	51 (64)	73	7 (9.6)	3 (4.1)	10 (14)	26 (36)	36 (49)

For each experimental condition, 2 to 4 replicates were performed. Chi Square test: Comparisons OTA-exposed versus vehicle control (1%MeOH): not significant

### Effects on embryo morphokinetics and blastocyst quality

At 10 μmol/L, the presence of OTA during maturation increased the rate of embryos arrested at the 2–4 cell stage (*p* < 0.01). At 0.1 μmol/L, OTA increased the rates of embryos arrested at the 8–16 cell stage (*p* < 0.05) and, correspondingly, it reduced the rates of embryos at the 4–8 cell stage (*p* < 0.05). At 0.1 nmol/L, it reduced the rates of embryos developed up to the 4–8 cell stage (*p* < 0.05) and to the 8–16 cell stage (*p* = 0.05). Despite these differences observed at specific stages of embryo development, the total cleavage and blastocyst formation rates did not change compared with controls, at any examined OTA concentration (Table 6). In Online Resource 4, photomicrographs representative of different embryo cleavage stages observed under phase contrast microscopy (1a–1e) and epifluorescence microscopy after fixation (2a–2e) are shown.

**Table 6:** Effects of in vitro oocyte exposure to OTA during IVM on embryo development

OTA concentration (μmol/L)	No. of cultured oocytes	No. of evaluated oocytes	Embryo developmental stages Fluorescence microscopy-based nuclear chromatin evaluation *N* (%)						
			2–4 cells	4–8 cells	8–16 cells	16–32 cells	Blastocyst	Total cleaved	Cleaved degenerated
0	225	171	13 (7.6) a	30 (17.5) a	24 (14) a	11 (6.4)	5 (2.9)	83 (48.5)	8 (4.6)
10	199	182	32 (17.6) c	21 (11.5)	18 (9.9)	5 (2.7)	9 (4.9)	86 (47.2)	2 (1.1)
1	125	103	8 (7.7)	16 (15.5)	21 (20.4)	9 (8.7)	3 (2.9)	57 (55)	9 (8.7)
0.1	127	93	4 (4.3)	7 (7.5) b	23 (24.7) b	2 (2.1)	4 (4.3)	40 (43)	9 (9.6)
0.01	125	95	12 (12.6)	14 (14.7)	8 (8.4)	5 (5.2)	4 (4.2)	43 (45)	8 (8.4)
0.001	100	73	7 (9.5)	18 (24.6)	10 (13.7)	5 (6.8)	1 (1.3)	41 (56)	5 (6.8)
0.0001	180	137	19 (14)	10 (7) b	9 (6.5) (*)	6 (4)	7 (5.1)	51 (37)	10 (7)

For each experimental condition, 5 to 10 replicates were performed. Chi square test: Comparisons OTA-exposed versus vehicle control (1%MeOH): a, b = *p* < 0.05; a, c = *p* < 0.01; (*) = *p* = 00549

**Fig. 4:**
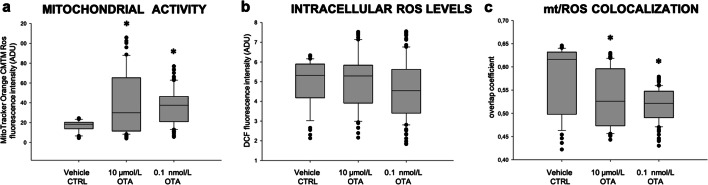
Boxplots representing mitochondrial membrane potential (panel **a**) and intracellular ROS levels (panel **b**) in control blastocysts and blastocyst derived from oocytes exposed to 10 μmol/L and 0.1 nmol/L OTA. Data are referred to blastocysts in Table 6. In panels **a** and **b**, values are presented as fluorescence intensities of MitoTracker Orange and DCF, respectively. In panel **c**, mitochondria/ROS colocalization is presented as overlap coefficient. One-way ANOVA Kruskal-Wallis non-parametric test, comparisons OTA-exposed vs control. *p < 0.05

**Fig. 5:**
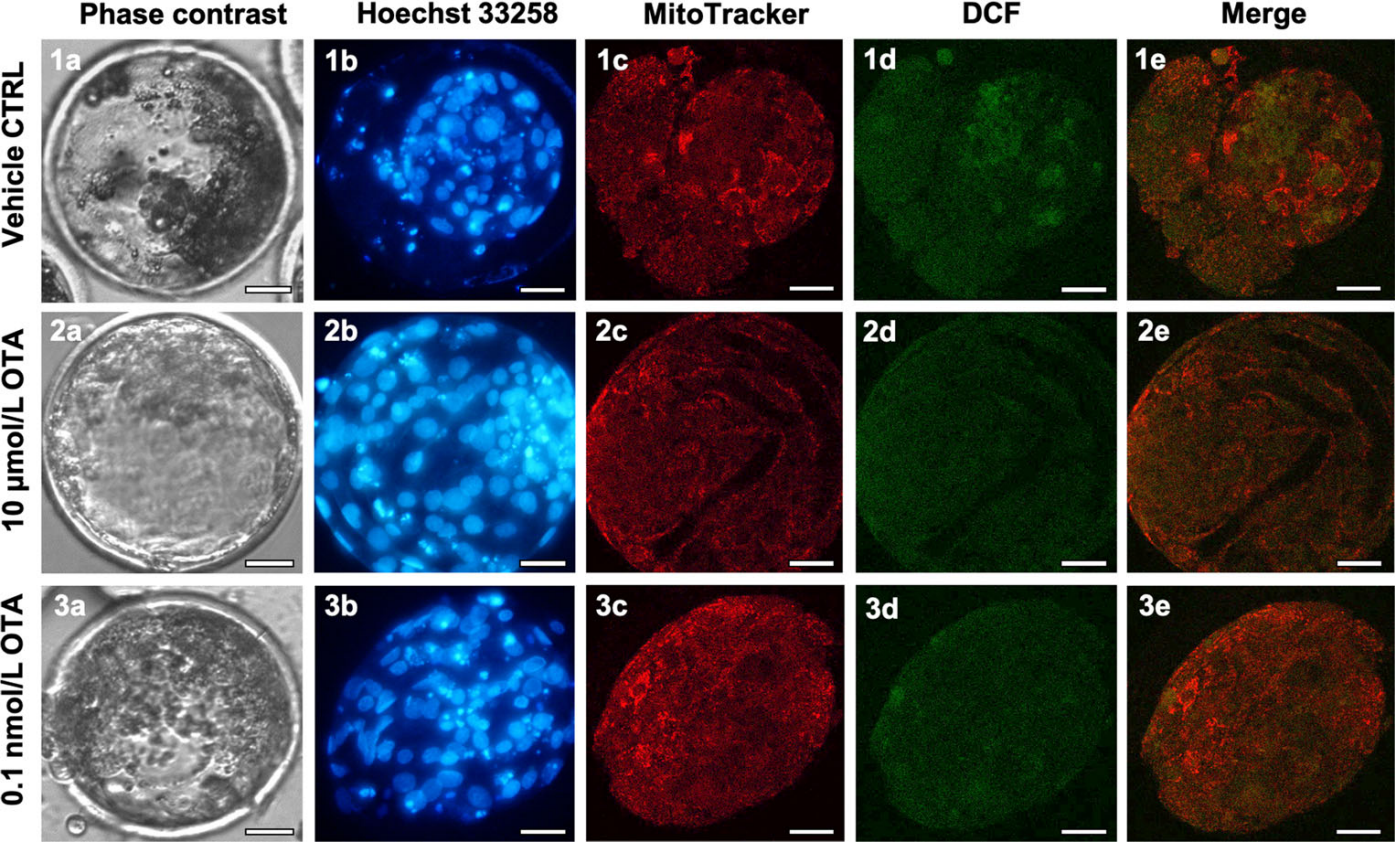
Photomicrographs showing representative images of a control blastocyst (lane 1) and of blastocysts obtained from oocytes exposed to 10 μmol/L (lane 2) and 0.1 nmol/L (lane 3) OTA, respectively. Images are referred to blastocysts in Table 6. MitoTracker Orange and DCF were used to label mitochondria and ROS, respectively. Nuclear chromatin was stained with Hoechst 33258. For each blastocyst, the corresponding phase-contrast images showing cell morphology (column a), epifluorescence images showing nuclear chromatin (column b) and confocal images showing the mitochondrial distribution pattern (column c), ROS localization (column d), mitochondria/ROS merge (column e) are shown. Scale bars represent 40 μm. DCF, dichlorodihydrofluorescein; ROS, reactive oxygen species

Blastocyst morphology, whether early, expanded, or hatched, was not affected by oocyte maturation in the presence of OTA. Actually, OTA tended to reduce the blastocyst expansion rate and, particularly at 10 μmol/L and 0.1 nmol/L, some blastocyst developed a barely visible blastocoelic cavity faster, but the differences did not attain statistical significance (Table 7). The total number of nuclei was not affected. However, as shown in Online Resource 5, the percentage of apoptotic nuclei was significantly higher after low OTA exposure (0.1 μmol/L to 0.1 nmol/L) compared with controls (Table 7). Blastocysts obtained in at least three runs/condition, under a micromolar (10 μmol/L) and a nanomolar concentration (0.1 nmol/L) were analyzed for bioenergetic and oxidative status and compared with controls. As a long-term effect of oocyte exposure during IVM, OTA significantly increased blastocyst mitochondrial membrane potential at both tested concentrations (*p* < 0.05; Fig. 4 panel **a**, and Fig. 5 column c). Intracellular ROS levels were not affected (Fig. 4 panel **b**, and Fig. 5, column d) and mitochondria/ROS colocalization was significantly reduced at both tested concentrations (*p* < 0.05; Fig. 4 panel **c**, and Fig. 5, column e).

**Table 7:** Effects of in vitro oocyte exposure to OTA during IVM on blastocyst quality

OTA concentration (μmol/L)	No. of evaluated blastocysts (*)	Blastocyst quality parameters *N* (%)			
		Expanded morphology *N* (%)	Number of nuclei (mean ± sd)	Apoptotic index (no. of apoptotic cells/blastocyst)	Range of apoptosis
0	5	4 (80)	53 ± 7.5	39/265 (15) a	(6.3–24)
10	9	5 (56)	58 ± 26	101/521 (19)	(9.6–46.9)
1	3	3 (100)	66 ± 6.5	27/199 (14)	(7.5–23)
0.1	4	3 (75)	57 ± 24.7	50/229 (22) b	(4.6–82)
0.01	4	4 (100)	56 ± 44.2	59/224 (26) c	(10.5–83)
0.001	1 (**)	0 (0)	28	24/28 (85)	(85)
0.0001	7	3 (43)	51 ± 21.5	116/357 (33) e	(6.8–71.9)

(*) Data are referred to blastocysts obtained in Table 6

Chi Square test: Comparisons OTA-exposed versus vehicle control (1%MeOH): a, b *p* < 0.05; a, c = *p* < 0.01; a, e *p* < 0.0001

(**) Data of this blastocyst were not statistically analyzed

**Fig. 6:**
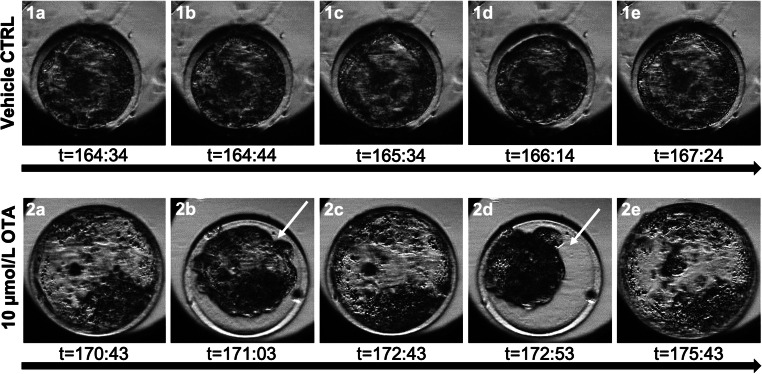
Photomicrographs showing representative contractions observed during time-lapse monitoring of in vitro cultured blastocysts obtained from oocytes matured in vitro in the absence (vehicle controls: lane 1; a–e) or presence (lane 2; a–e) of OTA. Starting from the end of day 7, control blastocysts underwent physiological small contractions preluding expansion whereas the OTA-blastocyst underwent abrupt sequential collapses. White arrows indicate blastocyst collapses. Images are referred to blastocysts in Table 8. See also videos 1 and 2 (Online Resource 6 and 7) showing TLM of representative embryos cultured under control and OTA treatment conditions, respectively

Due to the lack of effects at micromolar concentration on most of blastocyst morphofunctional parameters reported above, embryo morphokinetics at micromolar concentration was assessed. In the control group, 11/16 oocytes (69%) cleaved and in the OTA group, 7/16 oocytes (44%) cleaved. Mophokinetic parameters of these developing embryos were followed for 8 days. Three out of 11 control embryos (27%) and two out of 7 OTA embryos (29%) reached the blastocyst stage. Embryos in the OTA treatment showed significantly delayed times of cell cleavage at the 5-cell stage (t5; Table 8), indicating that OTA-embryos take significantly longer time to overcome the 4 cell stage and to undertake the 4–8 cell cleavage. The other cleavage times (*t*2, *t*3, *t*4, *t*8) and the time of blastocyst formation (*t*B) and blastocyst expansion (*t*Bexp) did not differ significantly between OTA and controls. A relevant delay was also observed for *t*8 after oocyte exposure to OTA, but it did not attain statistical significance (*p* = 0.05). OTA embryos did not progress to extruding cells through the zona (hatching blastocysts), so that in examined conditions, the time of blastocyst cell protrusion (tBcp) could not be recorded. It can be also seen that cc2 and s2, the two variables related to the duration of cell cycles, equivalent to t3-t2 and t4-t3, respectively, did not change between OTA-exposed group and controls (Table 8). Notably, in both OTA-blastocysts, serial collapse events occurred with volume reduction to less than 50%. The time in which the first collapse took place was indicated as tBlcoll. These episodes did not occur in control blastocysts in which, during the expansion phase, ritmically regular pulsatile small contractions occurred with volume reduction no more than 10%. Overall, since the tB to the end of embryo culture time, blastocysts underwent about 20 contractions. However, for OTA-derived blastocysts, some out of these contractions (7 for one and 3 for the other) occurred as collapses. In Fig. 6, sequential pictures (1a-1e) of a blastocyst developed after IVM in vehicle control, undergoing normal contractions and sequential pictures of a blastocyst developed after IVM under OTA treatment, undergoing collapse events (2a-2e) are shown.

**Table 8:** Effects of in vitro oocyte exposure to OTA on morpho-kinetic parameters of embryo development

Culture conditions	*t*2	*t*3	*t*4	*t*5	*t*8 (*)	*t*B	*t*B exp	*t*B coll	cc2 (*t*3-t2)	s2 (*t*4-*t*3)
CTRL (*n* = 11)	17.8 ± 10.1	24.8 ± 10.9	25.1 ± 9.4	29.2 ± 9.9 a	37.7 ± 13.8	153.8 ± 10.2	162.9 ± 1.2	/	8.2 ± 6.3	5.3 ± 5.1
OTA 10 μmol/L (*n* = 7)	17.8 ± 4.8	26.8 ± 7.5	28.7 ± 4.9	45.7 ± 18.7 b	65.2 ± 31.5	140.6 ± 14.0	156.8 ± 2.2	163.4 ± 1.6	10.5 ± 4.3	2.2 ± 3.2

For each experimental condition, 2 replicates were performed. Table legend: *B*, blastocyst; *B exp*., expanded blastocyst; *B coll*, first blastocyst collapse. *T* test: comparisons OTA-exposed versus vehicle control (1%MeOH): a, b = *p* < 0.05. (*) *p* = 0.05

## Discussion

The main interest for determining OTA toxicity on female fertility and early development is given by its wide diffusion in human food and animal feed and by previous evidence as a risk factor for abnormal embryonic development and teratogenic effects (Huang and Chan 2014). Due to current dietary regimens, including cereal-based feed, sheep are increasingly exposed to mycotoxins, thus playing a role as models for human (Baird 1983; Campbell et al. 2003; Cotterill et al. 2013; Ledda et al. 1997; Leoni et al. 2007, 2015). Moreover, the juvenile sheep model is of particular interest to study the effects of juvenile exposure to contaminants on female fertility, thus mirroring exposure during pediatric and adolescent age.

OTA induced a dose-dependent inhibitory effect on cumulus expansion and oocyte maturation. These data are in agreement with a previous study in porcine oocytes reporting that OTA disturbed cumulus and granulosa cell diffusion and proliferation (Lan et al. 2019). OTA affected oocyte maturation only at micromolar concentrations. These findings are in agreement with previously reported studies in mice (Huang and Chan 2014; Jia et al. 2019) and pigs (Lu et al. 2018). However, data on meiosis progression with effects on earlier meiotic stages were not reported in those studies. In another study in porcine oocytes, meiosis progression was followed at two culture times (27 h and 54 h). At the end of IVM, OTA was found to significantly reduce progression to the MII stage. This was demonstrated to be due to negative effects on spindle organization and chromosome alignment, possibly through the mitogen-activated protein kinase (MAPK) signalling pathway, that, from transcriptomic analysis, was found to be a major pathway affected by OTA (Lan et al. 2019). To the best of our knowledge, no studies to date analyzed the effects of nanomolar OTA concentrations on oocyte maturation.

At the highest tested micromolar concentration, OTA changed the cytoplasmic distribution of mitochondria, leading to structural change in the bioenergetic status. Moreover, at micromolar levels, OTA reduced mitochondrial activity and ROS generation, inducing functional damage. These results highlight multiple aspects of oocyte bioenergetic damage caused by OTA. Indeed, the CLSM-based multiparametric method for assessment of oocyte mitochondrial energy/redox status allows the combined analysis of qualitative and quantitative parameters able to distinguish severe damage (loss of cell viability, expressed as reduced mitochondrial activity and ROS generation) from mild functional damage (oxidative stress expressed as an adverse boosting of mitochondrial membrane potential leading to increased intracellular ROS levels). In porcine oocytes, OTA treatment led to mitochondrial clustering (Lan et al. (2019) rather than dispersion, as in our study, indicating possible age-related differences. Indeed, juvenile sheep oocytes have fewer developed cytoplasmic microfilament networks (Velilla et al. 2005), necessary for mitochondrial migration, which could be more sensitive to the mycotoxin. Oocytes exposed to micromolar OTA concentrations also exhibited damage to mitochondrial function, with reduced mitochondrial membrane potential and ROS generation ability. OTA-related ROS reduction was most likely due to functional mitochondrial damage since mitochondria are the major source of ROS (Cadenas and Davies 2000). In this case, our data are not in agreement with those of previous studies which reported increased ROS levels indicating oxidative stress (Lan et al. 2019; Jia et al. 2019). These discrepancies could be due to age-related differences. Indeed, adult oocytes are possibly more resistant to OTA-induced mitochondria functional damage whereas juvenile oocytes, used in our study, possibly have higher sensitivity to OTA due to less developed antioxidant defences (Jiao et al. 2013; Piras et al. 2019). Moreover, colocalization of active mitochondria and intracellular ROS, a putative marker of the oocyte welfare (Martino et al. 2012), decreased following OTA exposure, suggesting that ROS could have been displaced in different cytoplasmic areas, with consequent cell damage. These toxic effects on mitochondrial function and ROS generation ability may have affected oocyte maturation, subsequent fertilization and embryo development and quality. Altered expression of genes related to tricarboxylic acid cycle and complexes I, III, IV, and V of respiratory chain, upon OTA exposure, (Lan et al. 2019) further suggests mitochondria involvement in OTA-induced toxicity.

Results on the effects of OTA on fertilization obtained at micromolar concentrations are in agreement with those of two previous studies. Mouse oocytes exhibit a reduced ability to be fertilized by fresh sperm upon exposure to10 μmol/L OTA during IVM but not at 1 μmol/L (Huang and Chan 2014; Jia et al. 2019). Here we demonstrate that OTA affected multiple aspects of fertilization with different mechanisms elicited by switching from micromolar to nanomolar concentrations. At the highest tested concentration, it inhibited the fertilization rate and the percentage of PN3 zygotes, indicating severe damage of pronuclear DNA duplication and migration, with possible involvement of the DNA replication apparatus and/or cytoskeletal elements controlling PN migration. Similarly, OTA-exposed porcine oocytes exhibited altered expression of DNA duplication-related genes (Lan et al. 2019). At lower micromolar OTA concentration, the normal fertilization rate was reduced, but with no effects on PN morphology and migration whereas at nanomolar concentrations, OTA reduced the fertilization rate with formation of multipronucleated zygotes. Sperm-free IVF experiments demonstrated that OTA did not induce parthenogenesis. Most likely, oocytes exposed to low OTA concentrations could be more susceptible to polyspermy, which is induced by oolemmal depolarization via an influx of Na + ions and migration of cortical granules (review by Georgadakis et al. 2016). These data indicate that OTA, at nanomolar concentration, although not affecting oocyte nuclear maturation and bioenergetic/oxidative status, affects other mechanisms of oocyte cytoplasmic maturation. Indeed, previous studies, in other cell systems, reported that OTA affects multiple cell signalling pathways, such as Ca^2+^ signalling, pH- and energy-homeostasis, and mitogen-activated protein kinases (MAPKs) pathways (Gekle et al. 2005). All these pathways are involved in the regulation of oocyte maturation (Sánchez and Smitz 2012), suggesting that nanomolar OTA, while not inducing cytotoxic effects, could enter the cumulus-oocyte complex cells eliciting subtle but strong effects, such as alterations of cell signalling pathways. Indeed, porcine oocytes exhibit alterations of MAPK protein expression upon OTA treatment (Lan et al. (2019). Further studies are necessary to explore these effects.

Surprisingly, total embryo cleavage and blastocyst formation rates were not affected by oocyte exposure to OTA. This result was unexpected since mouse oocytes exposed to OTA during IVM exhibited impaired embryonic development (Huang and Chan 2014; Jia et al. 2019). A possible explanation for these discrepancies may lie in species-specific effects (Lu et al. 2018). At any tested concentration, OTA did not affect blastocyst morphology and growth and, increased the apoptotic index only at nanomolar concentrations. Huang and Chan 2014 observeded increased apoptosis in mouse blastocysts derived from 10-μM OTA-pretreated oocytes, again suggesting species-specific differences.

At both micro and nanomolar concentrations, OTA boosted blastocyst mitochondrial function and reduced mitochondria/ROS colocalization. Mouse blastocysts, exposed in vitro for 24 h to OTA during the morula/blastocyst transition exhibited loss of mitochondria membrane potential and increased ROS generation (Hsuuw et al. 2013; Huang et al. 2019). A possible explanation of discrepancies with our study could be the exposure phase. In fact, in the studies in the mouse, embryos were exposed to OTA during in vitro embryo culture which may have acted with immediate stronger effects, whereas in our study, OTA exposure occurred during IVM with lighter long-term effects on blastocysts.

Considering the lack of strong deleterious effects at micromolar concentrations, we evaluated whether any effect of OTA, at such concentrations, on embryo morphokinetics. Embryos from oocytes preincubated with 10 μmol/L OTA showed significantly altered 4–8 cell cycle length, a fundamental step of embryo development in which, in sheep, embryonic genome activation (EGA) takes place. Moreover, OTA-treated blastocysts showed impressive collapse events, with total volume reduction of more than 50%, not observed in control blastocysts, which exhibited regular physiological contractions. In human, blastocyst collapse is strongly related to lower implantation rate (Marcos et al. 2015) and pregnancy rate (Sciorio et al. 2019). These considerations are in agreement with the reported increased post-implantation resorption after exposure of oocytes to OTA during IVM (Huang and Chan 2014). These data on OTA effects on blastocyst quality, on one side undoubtedly indicate OTA-induced damage but, on the other hand, could be interpreted as attempts of the embryo to recover, with different mechanisms, its viability and morphology. Indeed, in our study, significant alterations of cell cycle length were observed at micromolar doses whereas increased apoptosis occurred at nanomolar doses. Altered cell cycle length and apoptosis have been reported as mechanisms responsible for gradual depletion of abnormal blastomeres, aneuploid, or with damaged chromatin (Bolton et al. 2016).

In conclusion, this study demonstrated that, in the juvenile sheep model, OTA at micromolar concentrations induced different effects on COCs and embryos than at nanomolar concentrations. These different effects demonstrate that, in the COC, nonspecific effects characterize OTA-toxicity due to high exposure levels, having minor toxicological relevance, whereas relevant nanomolar concentrations do not excert “classical” toxic action. Rather, they induce specific cell signalling-mediated effects without alterations of cell viability or integrity. Overall, the data are of interest for the animal production industry. Moreover, given the translational relevance of the juvenile sheep model, they are important for the assessment of OTA exposure to human female fertility.

## Electronic supplementary material

Online Resource 1 Culture media composition (DOCX 20 kb)

Online Resource 2 Effects of OTA on cumulus expansion and apoptosis. (panel a) Typical phase contrast photomicrographs of COCs exposed to OTA during 24 h IVM and observed under stereomicroscopy. COC expansion was inhibited in presence of OTA as assessed by cumulus morphology. Cumuli with continuous edges, consisting of cells in close contact each other, were seen in samples exposed to 10 and 1 μmol/L OTA, whereas in cumuli cultured in control conditions or in presence of 0.1 nmol/L OTA, the edges were discontinuous following cell detachment and production of a viscous extracellular matrix. Black scale bars represent 200 μm. (Panel b) Representative images of cumulus cells observed after IVM in presence of OTA and TUNEL assay. Merge of green and blue fluorescence are shown and were related to Alexa Fluor 488 and Hoechst 33258 staining, respectively. White arrows indicate TUNEL positive cumulus cells (green fluorescence). White scale bars represent 10 μm. Numbers of analysed cumulus cells per experimental condition are indicated in Table 1. (PNG 1887 kb)

Online Resource 3 Photomicrographs showing typical nuclear chromatin configuration of oocytes in different meiotic stages obtained after IVM in presence or absence of OTA. Scale bars represent 40 μm. GV = Germinal Vesicle; MI = Metaphase I; TI = Telophase; MII = Metaphase II. (PNG 2491 kb)

Online Resource 4 Photomicrographs representative of different embryo cleavage stages observed under phase contrast microscopy (lane 1; a-e) and epifluorescence microscopy after fixation (lane 2; a-e). Scale bars represent 40 μm. (PNG 1756 kb)

Online Resource 5 Photomicrographs representative of blastocysts obtained after IVM in presence of OTA and observed as fresh (a) or after fixing and Hoechst 33258 staining (b-h). Blastocysts derived from oocytes exposed during IVM to lower OTA concentrations (from 0.1 μmol/L to 0.1 nmol/L) showed increased apoptotic index (see Table 7). Arrows indicate apoptotic nuclei. Scale bars represent 40 μm. OTA = Ochratoxin A; IVM = in vitro maturation. *p < 0.05. (PNG 2454 kb)

ESM 6 (AVI 7911 kb)

ESM 7 (AVI 8876 kb)

## Abbreviation

CLSM: Confocal laser scanning microscope
COC: Cumulus-oocyte complex
DCF: Dichlorofluorescein
GV: Germinal vesicle
IVF: In vitro fertilization
IVM: In vitro maturation
MII: Metaphase II
PB: Polar body
PN: Pronucleus
ROS: Reactive oxygen species
TI: Telophase I
