# Supplementary material for: Ochratoxin A affects oocyte maturation and subsequent embryo developmental dynamics in the juvenile sheep model
Source: Mycotoxin Res. 2020 Sep 29;37(1):23–37. doi: 10.1007/s12550-020-00410-y (PMC7819917; doi:10.1007/s12550-020-00410-y)
Supplement: Supplementary file 1 — Culture media composition (DOCX 20 kb) [file 12550_2020_410_MOESM1_ESM.docx]

**Ochratoxin A affects oocyte maturation and subsequent embryo developmental dynamics in the juvenile sheep model**

Journal name: Mycotoxin Research

Maria Elena Dell’Aquila^*^, Shafaq Asif, Letizia Temerario, Antonella Mastrorocco, Giuseppina Marzano, Nicola Antonio Martino, Giovanni Michele Lacalandra, Bernard AJ Roelen, Augusto Carluccio, Domenico Robbe, Fiorenza Minervini.

**Author for correspondence**

***** Maria Elena Dell’Aquila

Dept Biosciences, Biotechnologies and Biopharmaceutics, University of Bari Aldo Moro, Italy

E-mail: [mariaelena.dellaquila@uniba.it](mailto:mariaelena.dellaquila@uniba.it)

**Online Resource 1.** Medium composition

**IVM medium composition (Mastrorocco et al., 2019)**

| **PRODUCT** | **COMPANY**  **AND CODE** | **CONCENTRATION** |
| --- | --- | --- |
| TCM‐199 medium with Earle’s salts | Sigma M-4530 | basic medium |
| HEPES | Sigma H-4043 | 5.87 mmol/L |
| Sodium bicarbonate | Sigma S-5761 | 33.09 mmol/L |
| L‐glutamine | Sigma G-7513 | 0.1 g/L |
| Sodium pyruvate | Sigma P-2256 | 2.27 mmol/L |
| calcium lactate pentahydrate  dissolved in Milli Q water | Sigma L-4388  Gibco 15230001 | Calcium 1.62 mmol/L;  Lactate 3.9 mmol/L |
| Gentamicin | Sigma G-1272 | 50 µg/ml |
| Fetal Calf Serum | Sigma F-4135 | 20% (vol/vol) |
| ovine Follicle Stimulating Hormone (FSH) (*) | Sigma F-4520 | 10 µg/ml |
| ovine Luteinizing Hormone (LH) (*) | Sigma L-5269 | 20 µg/ml |
| Pluset (FSH+LH) (**) | Serono Pluset (***) | 10 µg/ml |
| 17 beta Estradiol  dissolved in pure Ethanol | Sigma E-2257  Sigma 443611 | 1 µg/ml |

(*) used in IVM experiments (Tables 1 and 2)

(**) used in IVF experiments and in vitro embryo culture experiments (Tables 3-8)

(***) De Oliveira-Santos et al., 2016

**IVF medium (SOF) composition (Mastrorocco et al., 2019)**

**adapted from Tervit et al., 1972 and Bogliolo et al., 2011**

| **PRODUCT** | **COMPANY AND CODE** | **CONCENTRATION** |
| --- | --- | --- |
| NaCl | Sigma S-7653 | 107.70 mmol/L |
| KCl | Sigma P-5405 | 7.16 mmol/L |
| KH_2_PO_4_ | Sigma P-8416 | 1.19 mmol/L |
| CaCl_2_-2H_2_O | Sigma C-7902 | 1.71 mmol/L |
| MgSO_4_-7H_2_O | Sigma M-5921 | 0,738 mmol/L |
| NaHCO_3_ | Sigma S-5761 | 25.07 mmol/L |
| Sodium Lactate | Sigma L-7900 | 3.30 mmol/L |
| Sodium Pyruvate | Sigma P-2256 | 0.33 mmol/L |
| Penicillin (sodium salt) | Sigma P-3032 | 100 units |
| Phenol Red | Sigma P-3532 | 2-3 grains |
| Heparin | Sigma H-3149 | 1 µg/ml |
| Milli Q water | Gibco 15230001 |  |
| Estrous sheep serum (ESS) | (*) | 2% (vol/vol) |

(*) Barrera et al., 2018

**In vitro embryo culture medium (SOF-aa) composition (Mastrorocco et al., 2019)**

**adapted from Walker et al., 1996, Bogliolo et al., 2011 and Martino et al., 2016**

| **PRODUCT** | **COMPANY AND CODE** | **CONCENTRATION** |
| --- | --- | --- |
| SOF as for IVF medium |  |  |
| Bovine Serum Albumin | Sigma A-9418 | 0.4& |
| Arginine | Sigma A-8094 | 100 µmol/L |
| Aspartic acid | Sigma A-7219 | 20 µmol/L |
| Asparagine | Sigma A-4159 | 20 µmol/L |
| Alanine | Sigma A-7469) | 500 µmol/L |
| Cysteine | Sigma C-7602 | 50 µmol/L |
| Glutamic acid | Sigma G-8415 | 50 µmol/L |
| Glutamine | Sigma G-8540 | 210 µmol/L |
| Glycine | Sigma G.8790 | 1.5 mmol/L |
| Histidine | Sigma H-6034 | 50 µmol/L |
| Isoleucine | Sigma I-7403 | 100 µmol/L |
| Leucine | Sigma L-8912 | 200 µmol/L |
| Lysine | Sigma L-5501 | 220 µmol/L |
| Methionine | Sigma M-5308 | 50 µmol/L |
| Ornithine | Sigma O-6503 | 20 µmol/L |
| Phenylalanine | Sigma P-5482 | 100 µmol/L |
| Proline | Sigma P-5607 | 50 µmol/L |
| Serine | Sigma S-4311 | 10 µmol/L |
| Taurine | Sigma T-8691 | 50 µmol/L |
| Threonine | Sigma T-8441 | 10 µmol/L |
| Tyrosine | Sigma T-8566 | 110 µmol/L |
| Valine | Sigma V-0513 | 270 µmol/L |
