# Supplementary figures and images for: Ochratoxin A affects oocyte maturation and subsequent embryo developmental dynamics in the juvenile sheep model
Source: Mycotoxin Res. 2020 Sep 29;37(1):23–37. doi: 10.1007/s12550-020-00410-y (PMC7819917; doi:10.1007/s12550-020-00410-y)

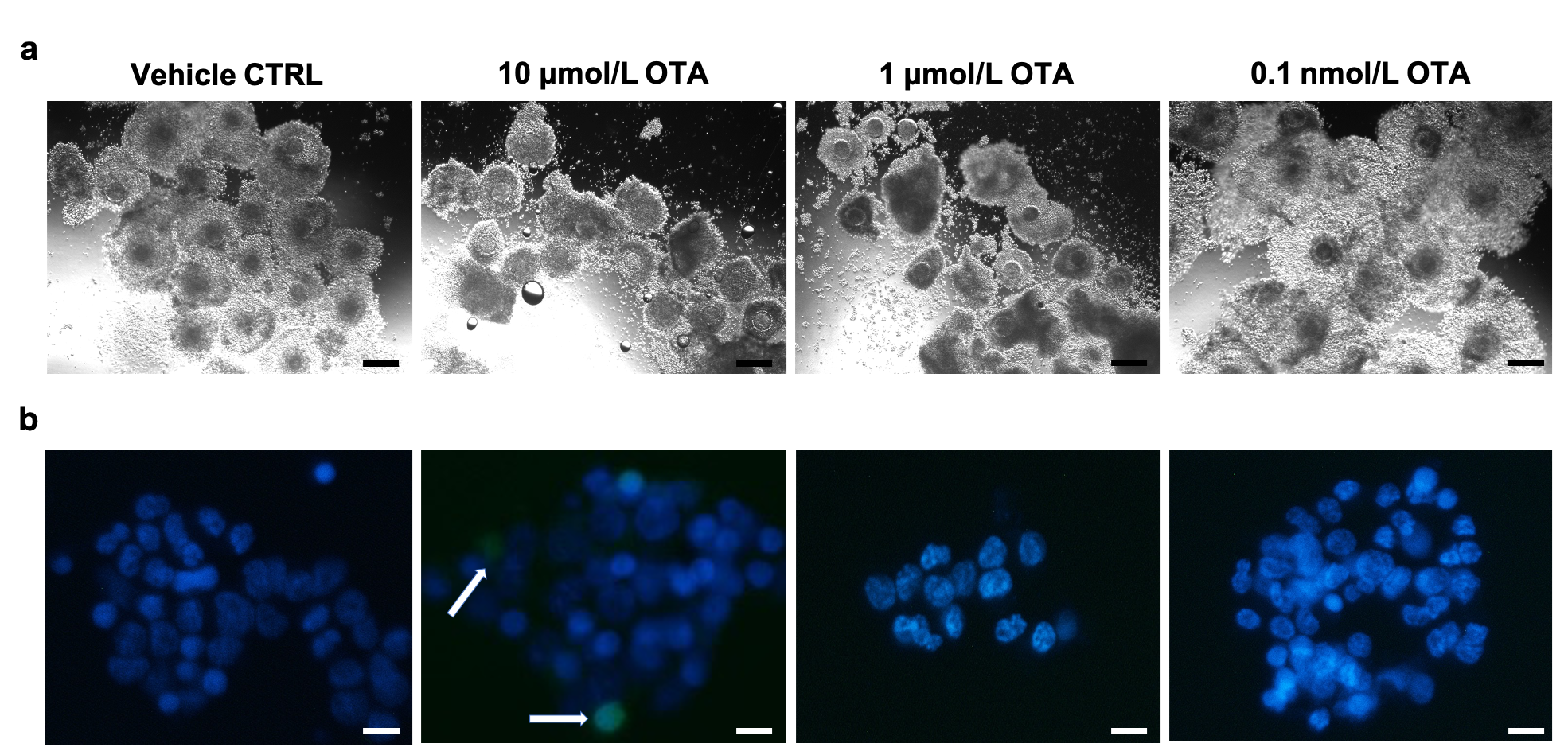

Supplement: Supplementary file 2 — Effects of OTA on cumulus expansion and apoptosis. (panel a) Typical phase contrast photomicrographs of COCs exposed to OTA during 24 h IVM and observed under stereomicroscopy. COC expansion was inhibited in presence of OTA as assessed by cumulus morphology. Cumuli with continuous edges, consisting of cells in close contact each other, were seen in samples exposed to 10 and 1 μmol/L OTA, whereas in cumuli cultured in control conditions or in presence of 0.1 nmol/L OTA, the edges were discontinuous following cell detachment and production of a viscous extracellular matrix. Black scale bars represent 200 μm. (Panel b) Representative images of cumulus cells observed after IVM in presence of OTA and TUNEL assay. Merge of green and blue fluorescence are shown and were related to Alexa Fluor 488 and Hoechst 33258 staining, respectively. White arrows indicate TUNEL positive cumulus cells (green fluorescence). White scale bars represent 10 μm. Numbers of analysed cumulus cells per experimental condition are indicated in Table 1. (PNG 1887 kb) [file 12550_2020_410_MOESM2_ESM.png]

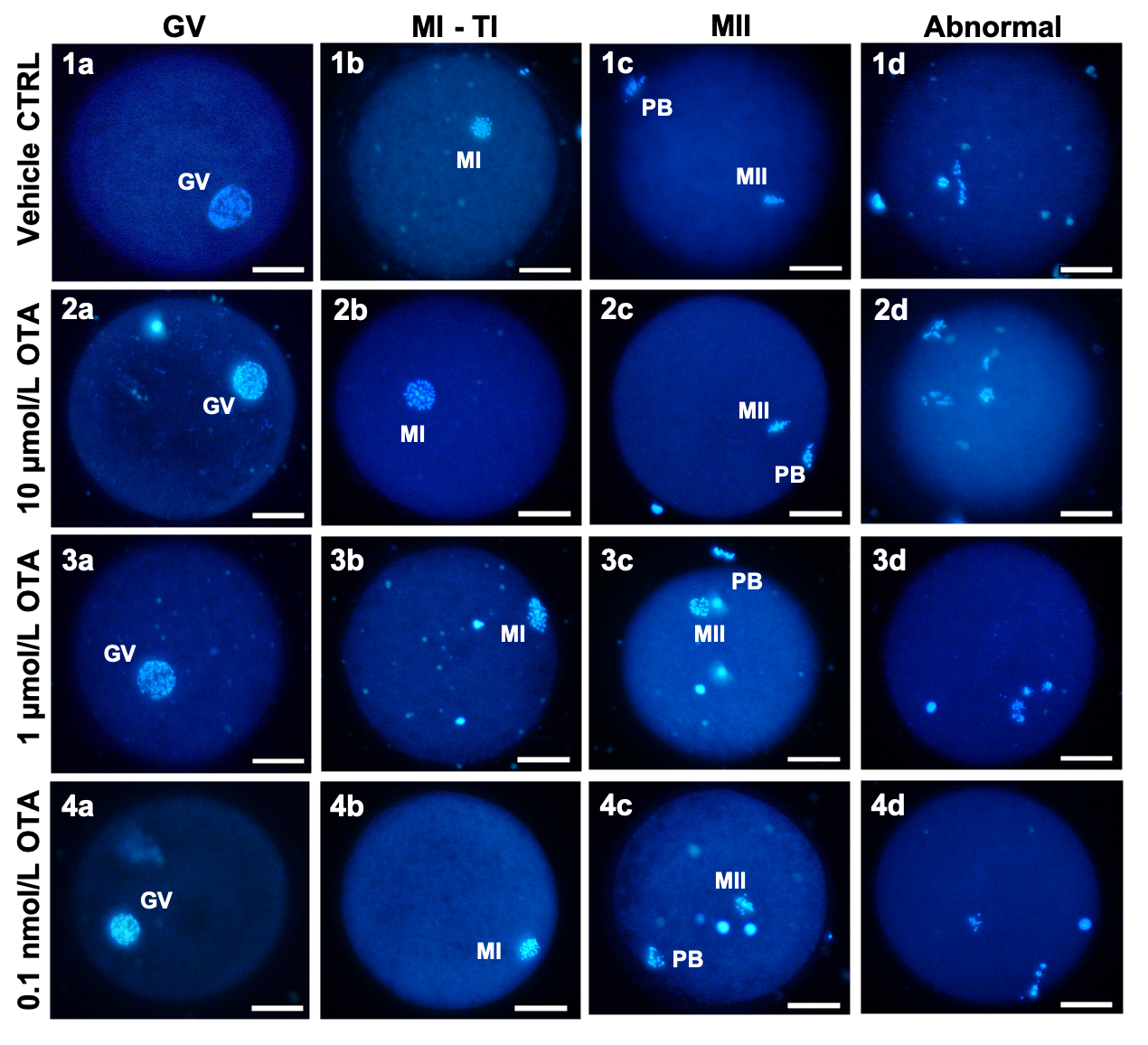

Supplement: Supplementary file 3 — Photomicrographs showing typical nuclear chromatin configuration of oocytes in different meiotic stages obtained after IVM in presence or absence of OTA. Scale bars represent 40 μm. GV = Germinal Vesicle; MI = Metaphase I; TI = Telophase; MII = Metaphase II. (PNG 2491 kb) [file 12550_2020_410_MOESM3_ESM.png]

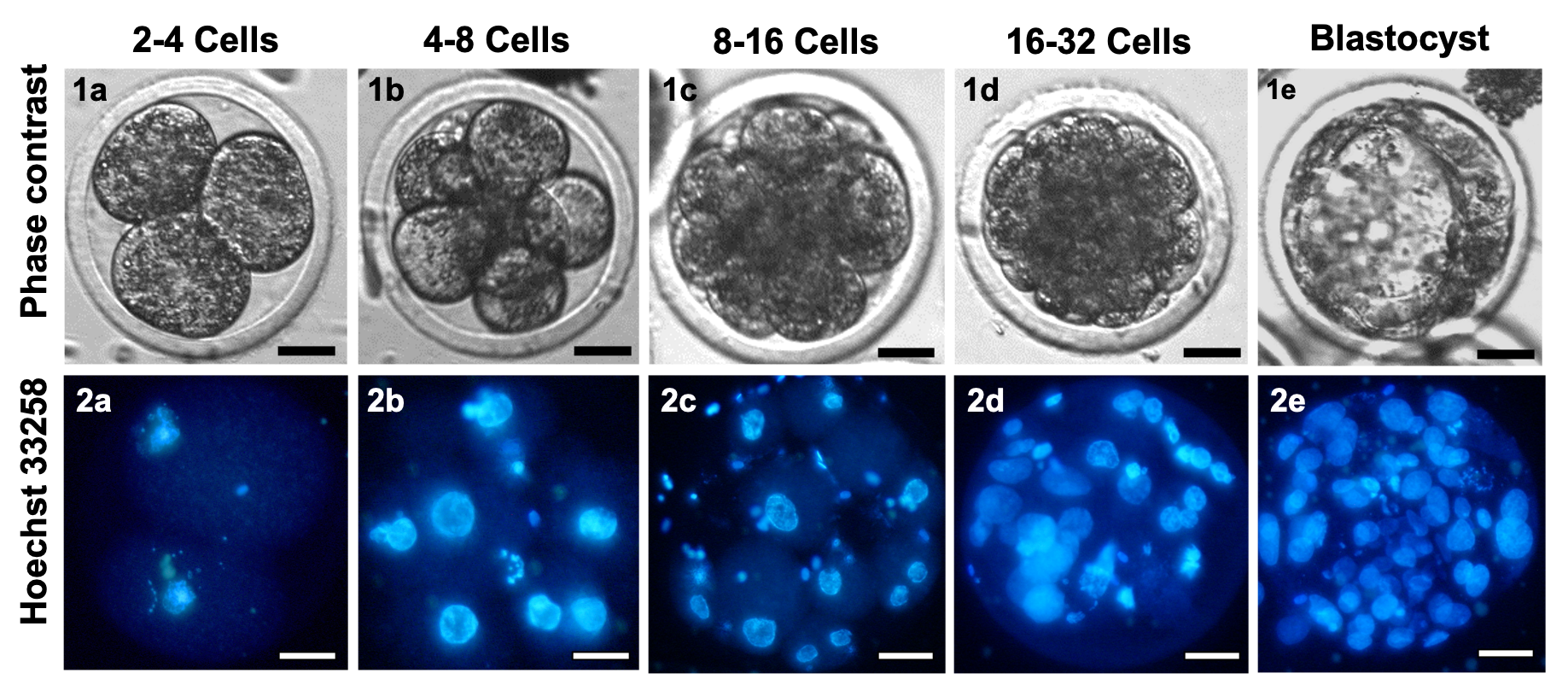

Supplement: Supplementary file 4 — Photomicrographs representative of different embryo cleavage stages observed under phase contrast microscopy (lane 1; a-e) and epifluorescence microscopy after fixation (lane 2; a-e). Scale bars represent 40 μm. (PNG 1756 kb) [file 12550_2020_410_MOESM4_ESM.png]

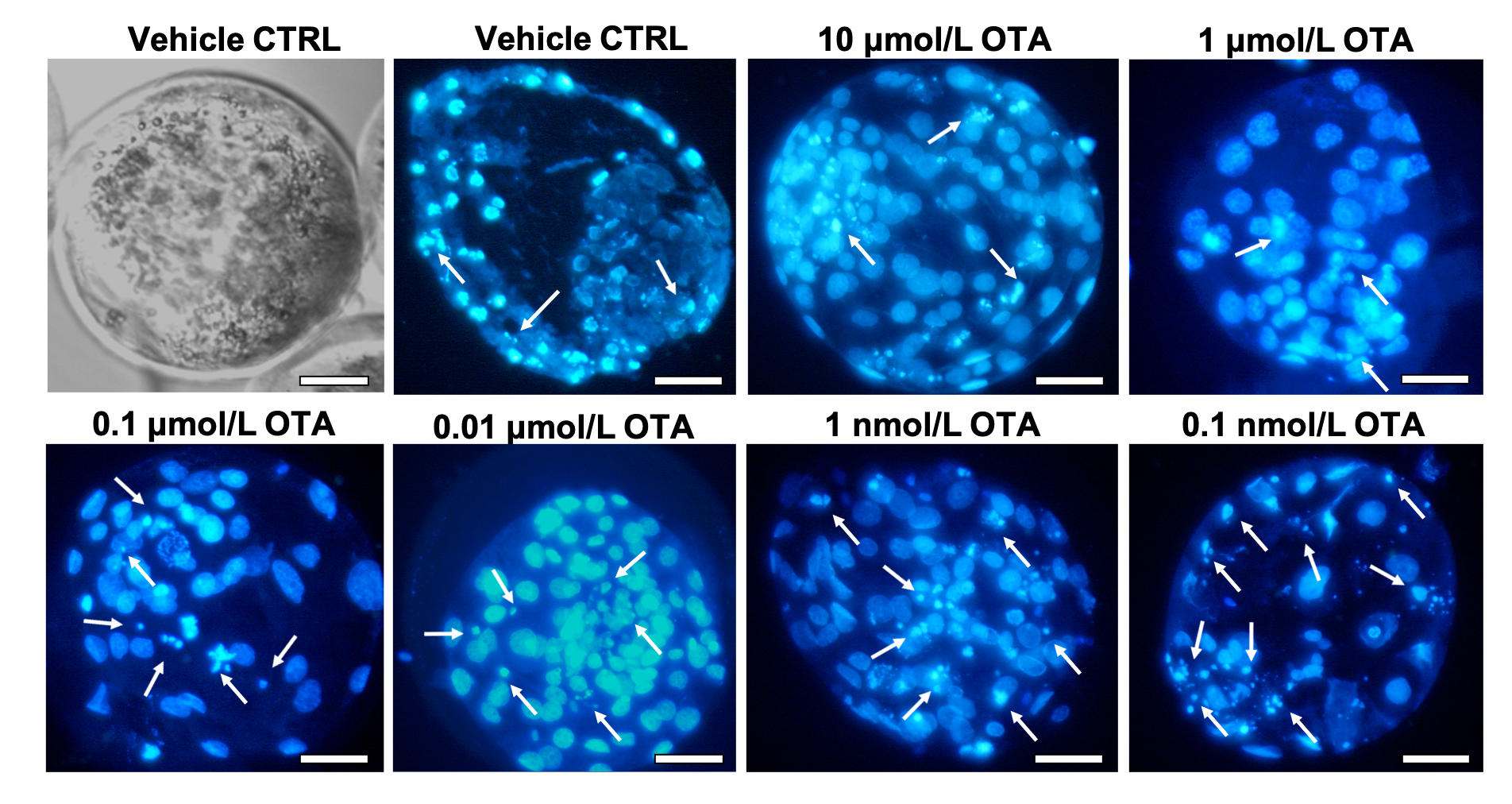

Supplement: Supplementary file 5 — Photomicrographs representative of blastocysts obtained after IVM in presence of OTA and observed as fresh (a) or after fixing and Hoechst 33258 staining (b-h). Blastocysts derived from oocytes exposed during IVM to lower OTA concentrations (from 0.1 μmol/L to 0.1 nmol/L) showed increased apoptotic index (see Table 7). Arrows indicate apoptotic nuclei. Scale bars represent 40 μm. OTA = Ochratoxin A; IVM = in vitro maturation. *p < 0.05. (PNG 2454 kb) [file 12550_2020_410_MOESM5_ESM.png]
